# Supplementary material for: Distinctiveness of Femoral and Acetabular Mesenchymal Stem and Progenitor Populations in Patients with Primary and Secondary Hip Osteoarthritis Due to Developmental Dysplasia
Source: Int J Mol Sci. 2024 May 9;25(10):5173. doi: 10.3390/ijms25105173 (PMC11121609; doi:10.3390/ijms25105173)
Supplement: Supplementary file 1 [file ijms-25-05173-s001.zip › ijms-2987884-supplementary.pdf]

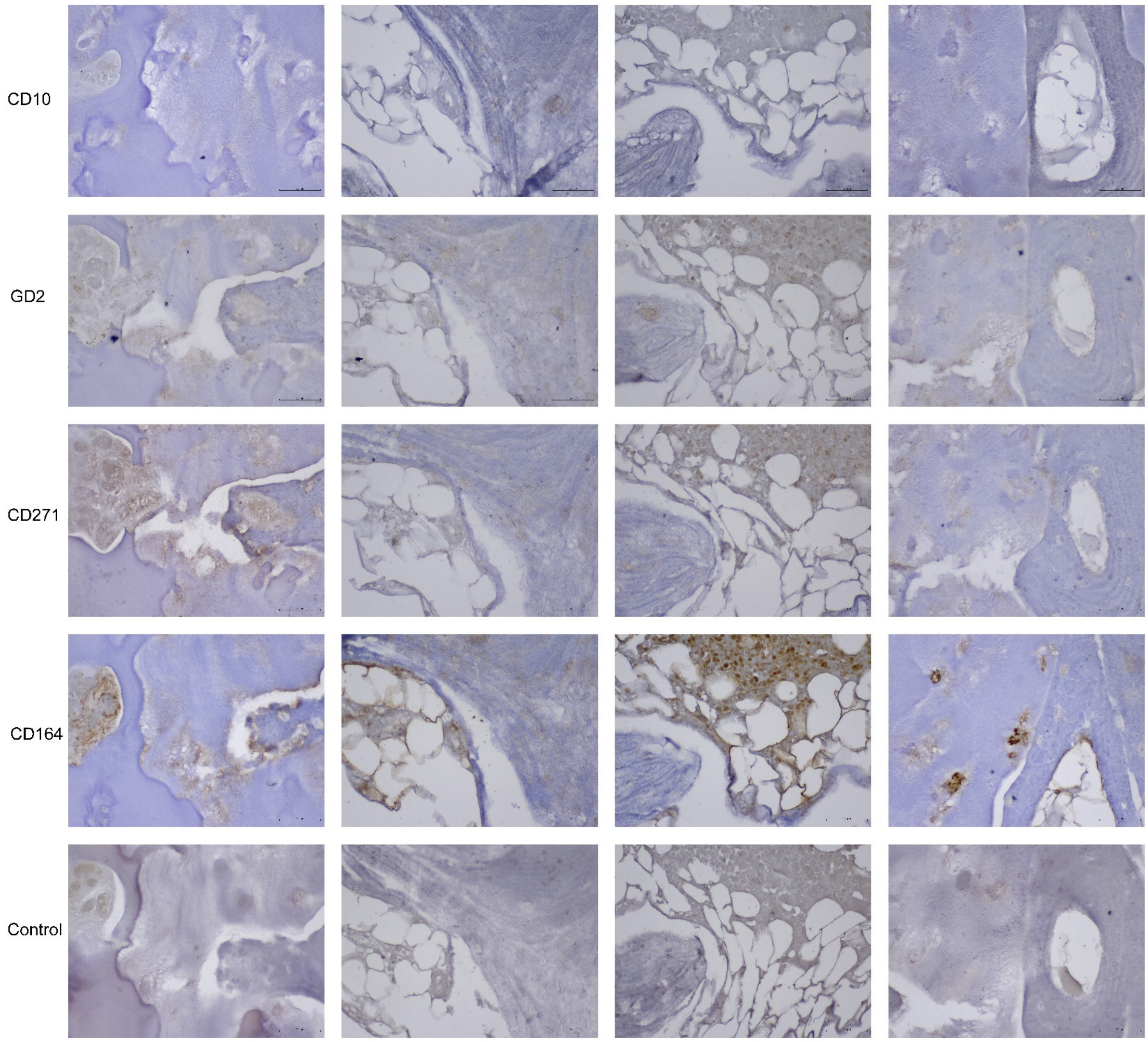

Figure S1. MSPC marker expression *in vivo* on secondary osteoarthritis due to developmental dysplasia of the hips acetabulum samples from patient No 2.

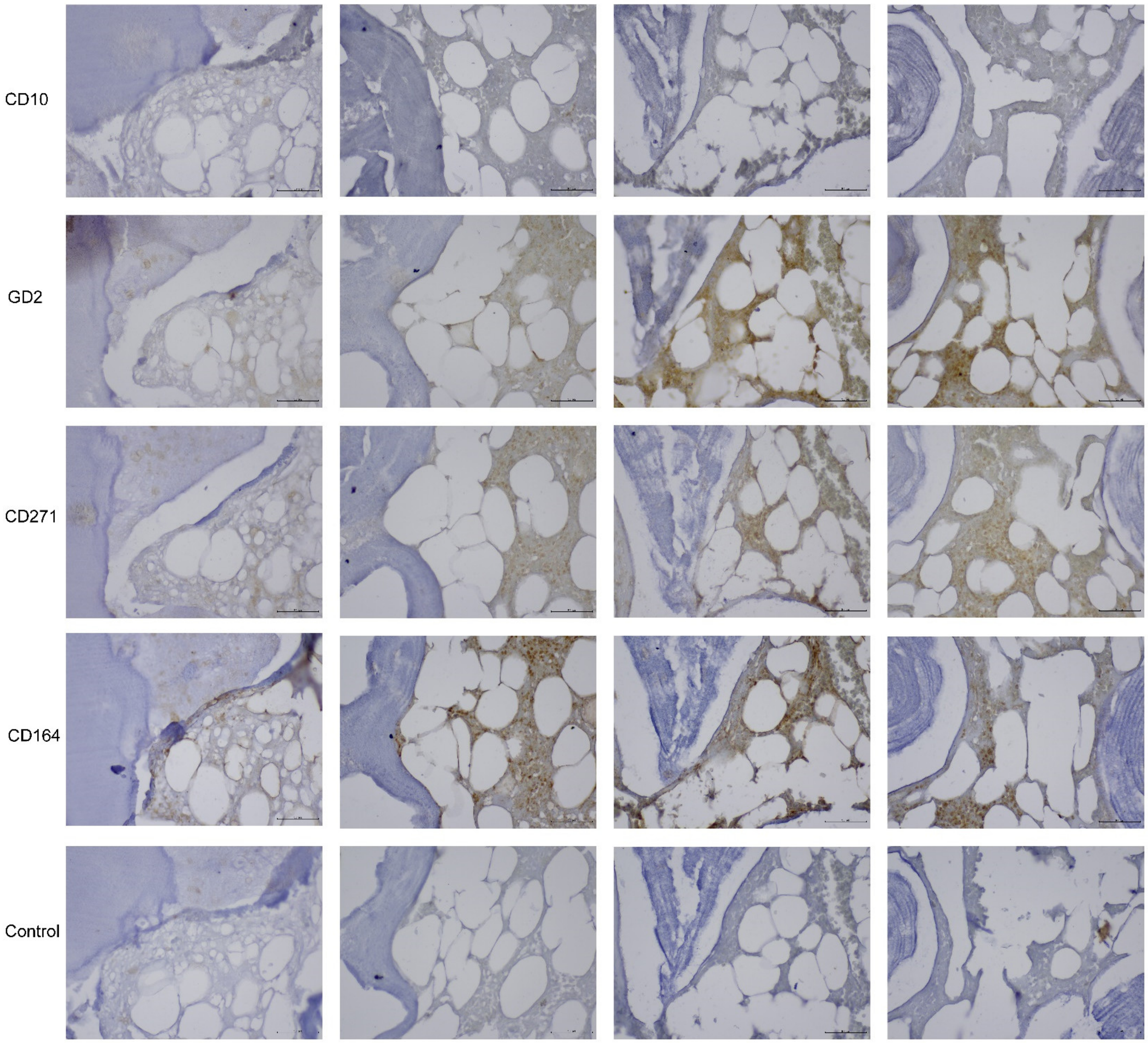

Figure S2. MSPC marker expression *in vivo* on secondary osteoarthritis due to developmental dysplasia of the hips femoral head samples from patient No 2.

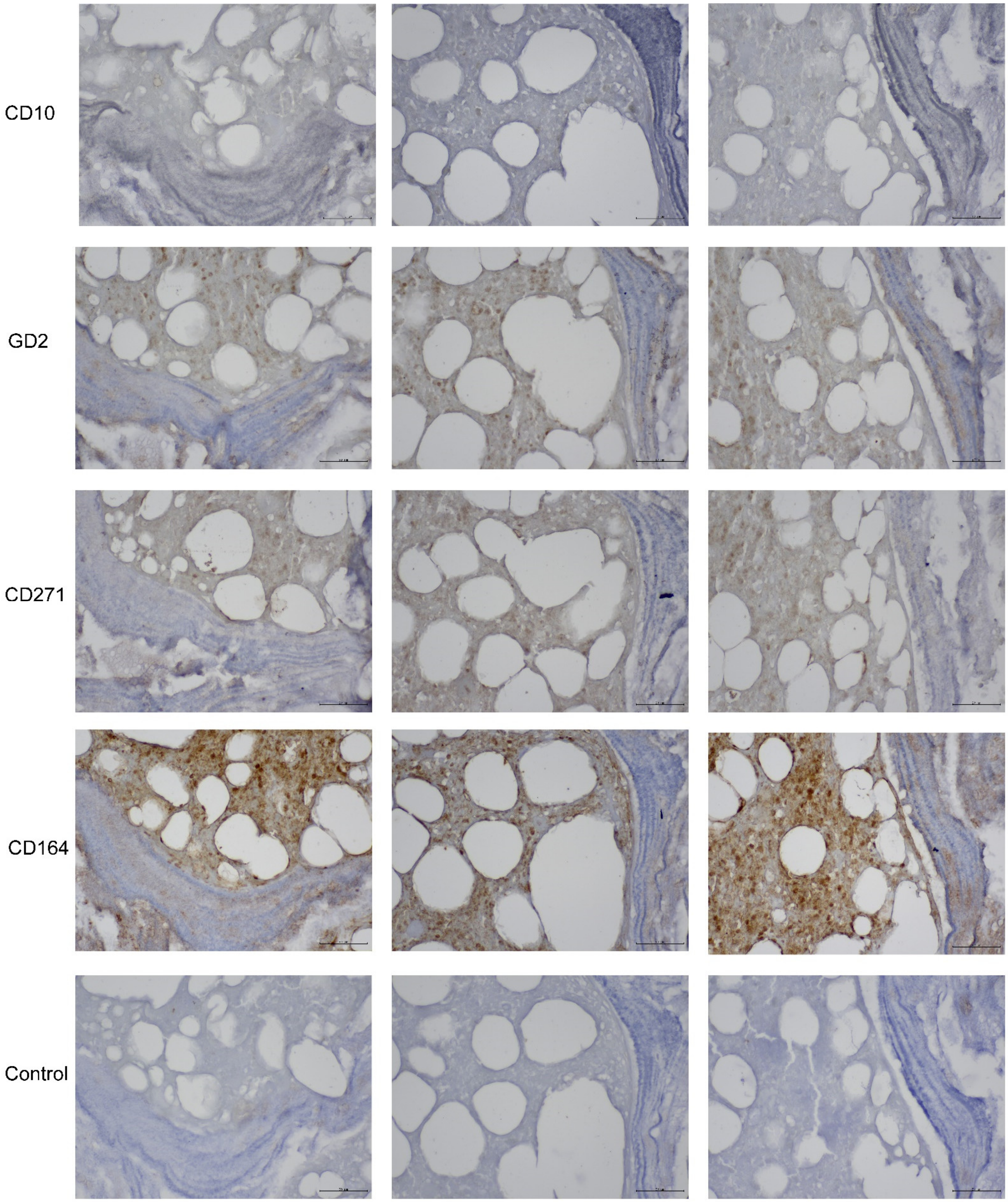

Figure S3. MSPC marker expression *in vivo* on secondary osteoarthritis due to developmental dysplasia of the hips acetabulum samples from patient No 7.

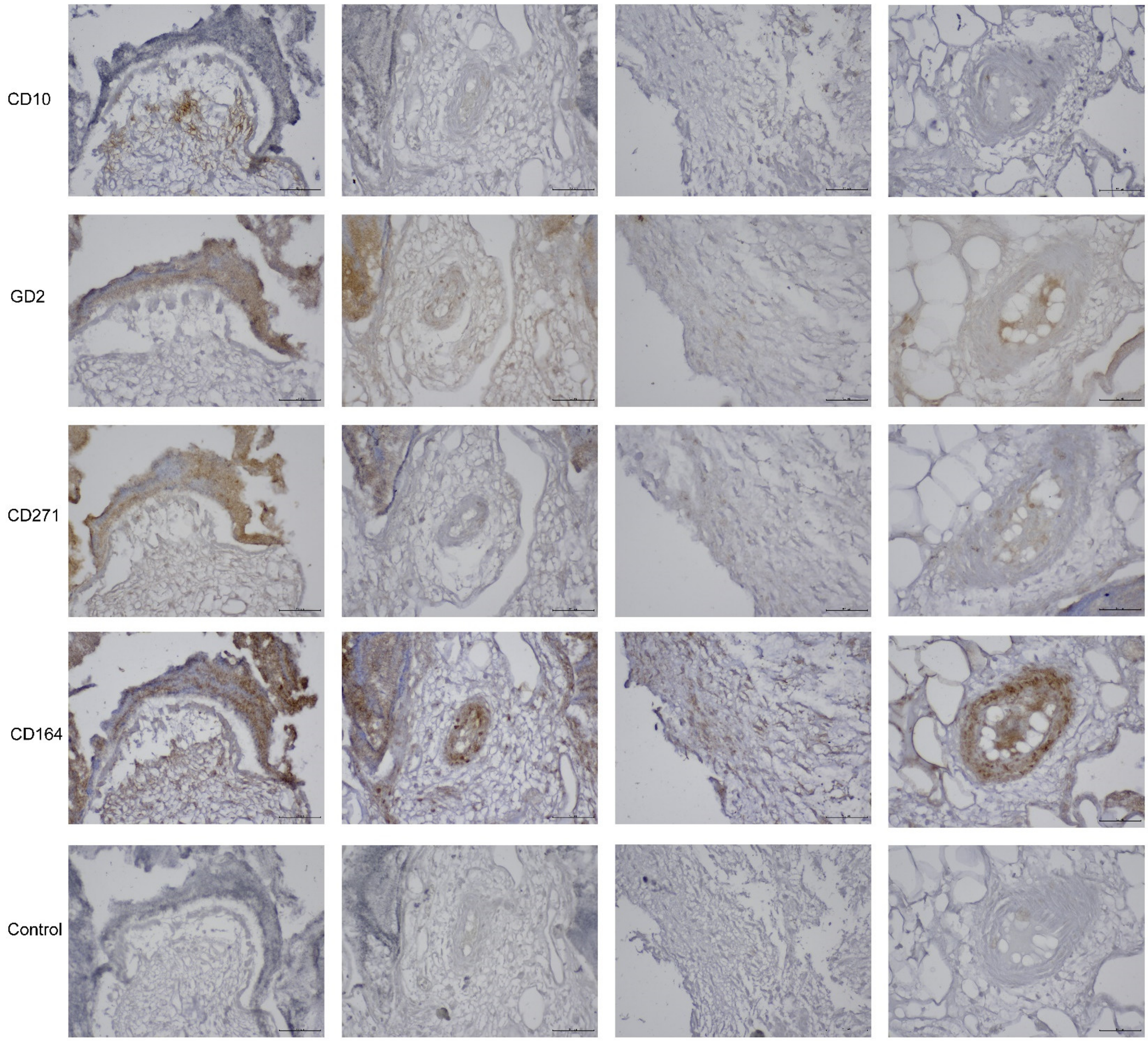

Figure S4. MSPC marker expression *in vivo* on secondary osteoarthritis due to developmental dysplasia of the hips femoral head samples from patient No 7.

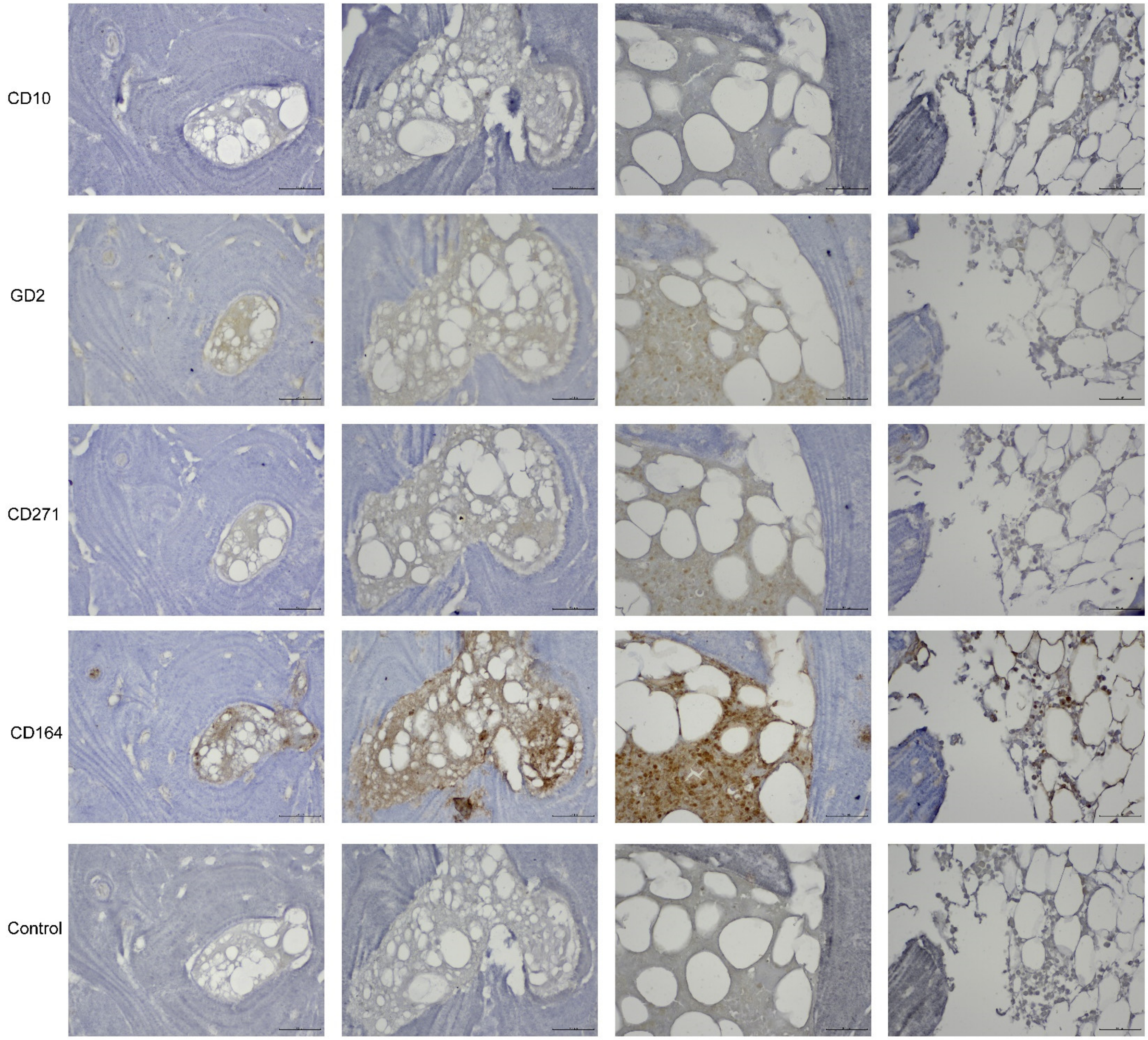

Figure S5. MSPC marker expression *in vivo* on secondary osteoarthritis due to developmental dysplasia of the hips acetabulum samples from patient No 11.

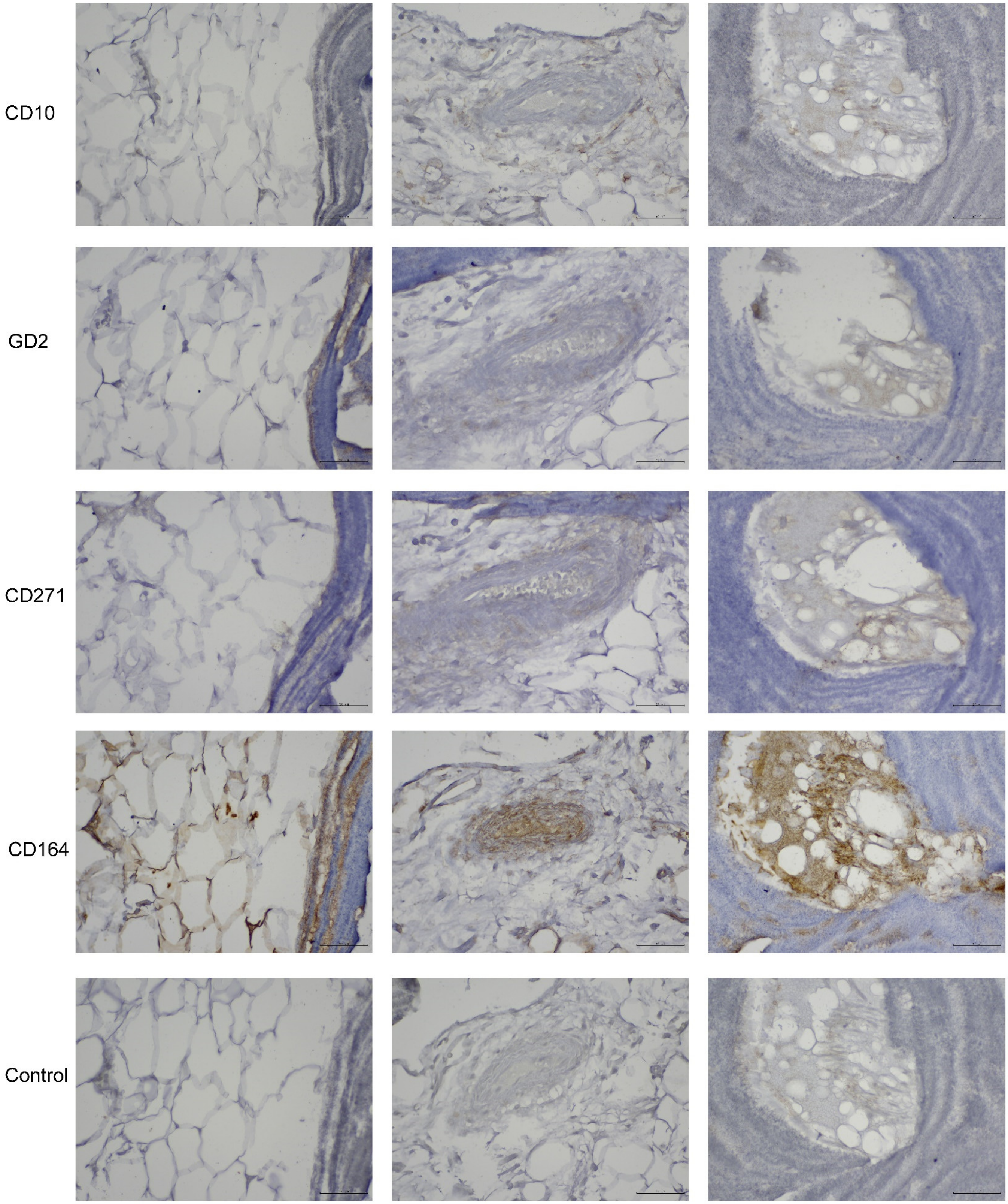

Figure S6. MSPC marker expression *in vivo* on secondary osteoarthritis due to developmental dysplasia of the hips femoral head samples from patient No 11.

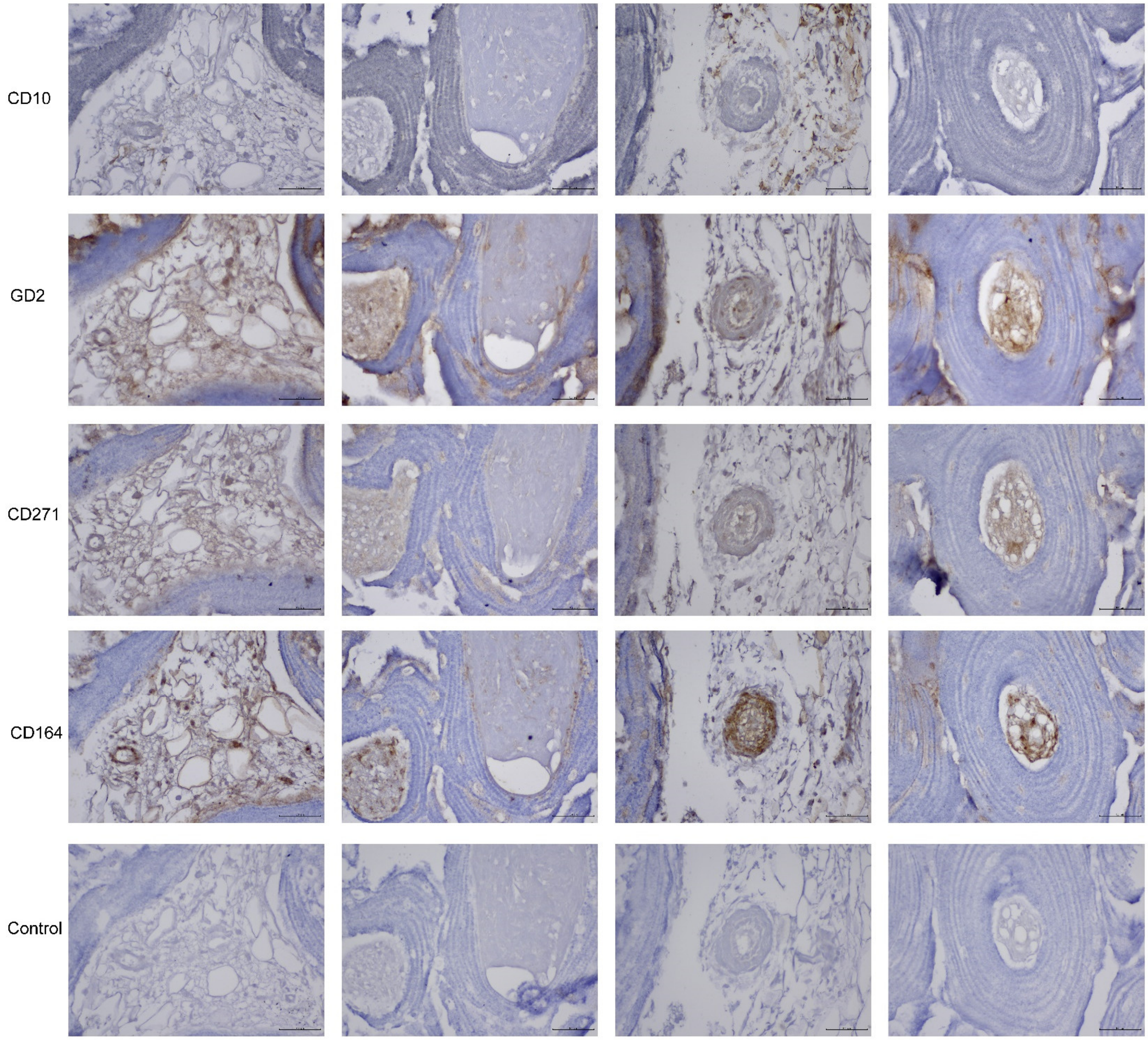

Figure S7. MSPC marker expression *in vivo* on primary hip osteoarthritis acetabulum samples from patient No 2.

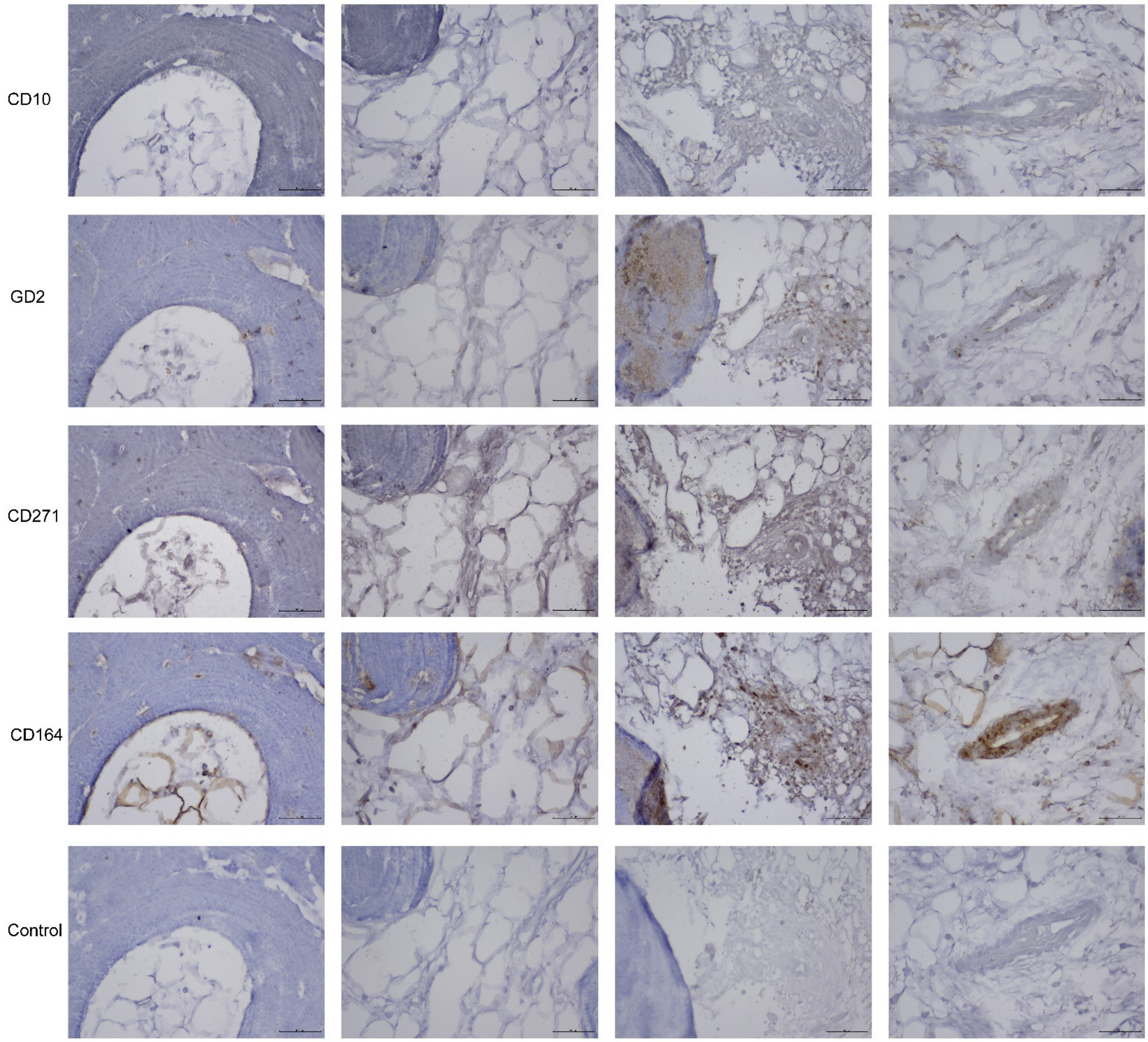

Figure S8. MSPC marker expression *in vivo* on primary hip osteoarthritis femoral head samples from patient No 2.

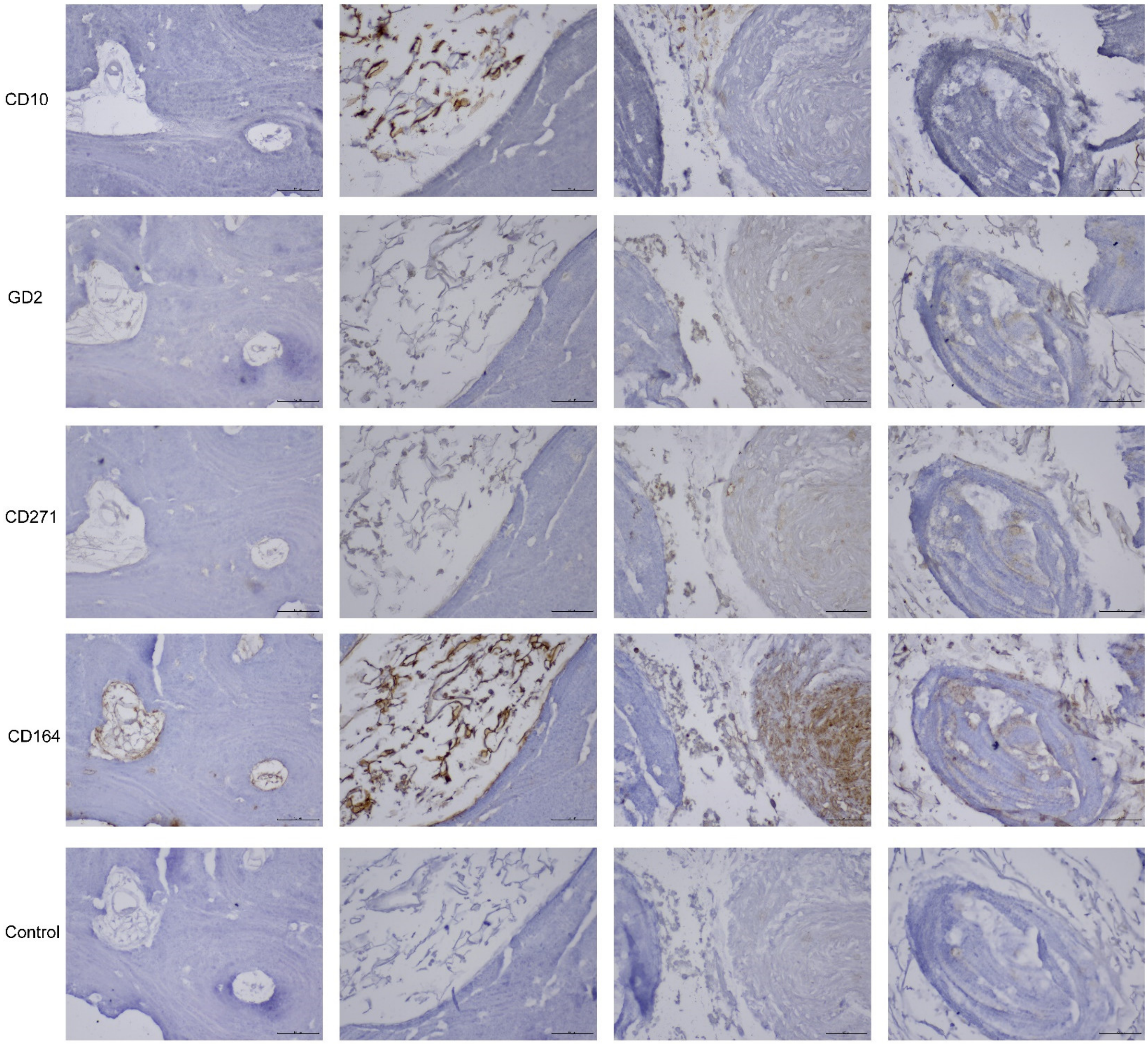

Figure S9. MSPC marker expression *in vivo* on primary hip osteoarthritis acetabulum samples from patient No 7.

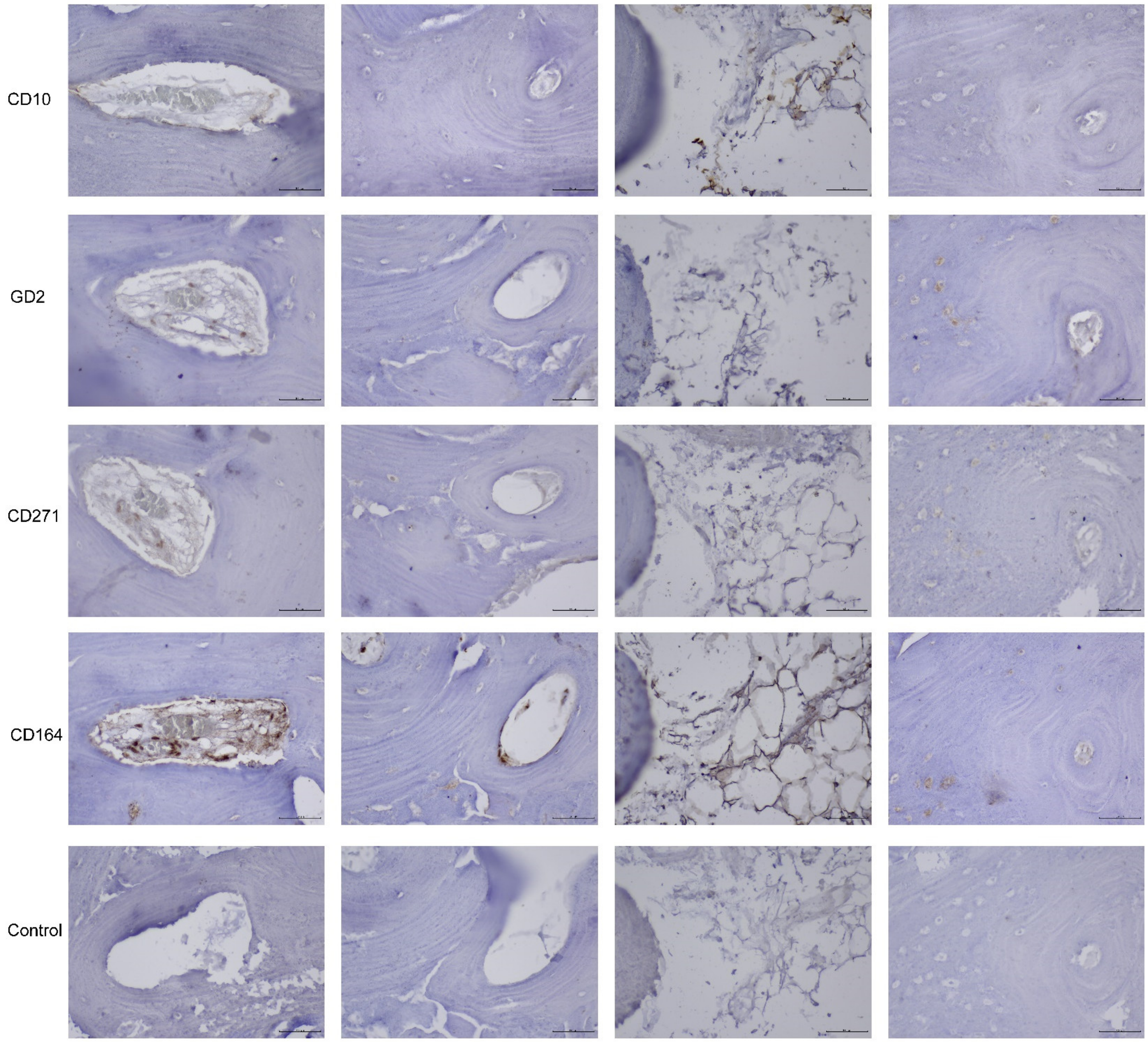

Figure S10. MSPC marker expression *in vivo* on primary hip osteoarthritis femoral head samples from patient No 7.

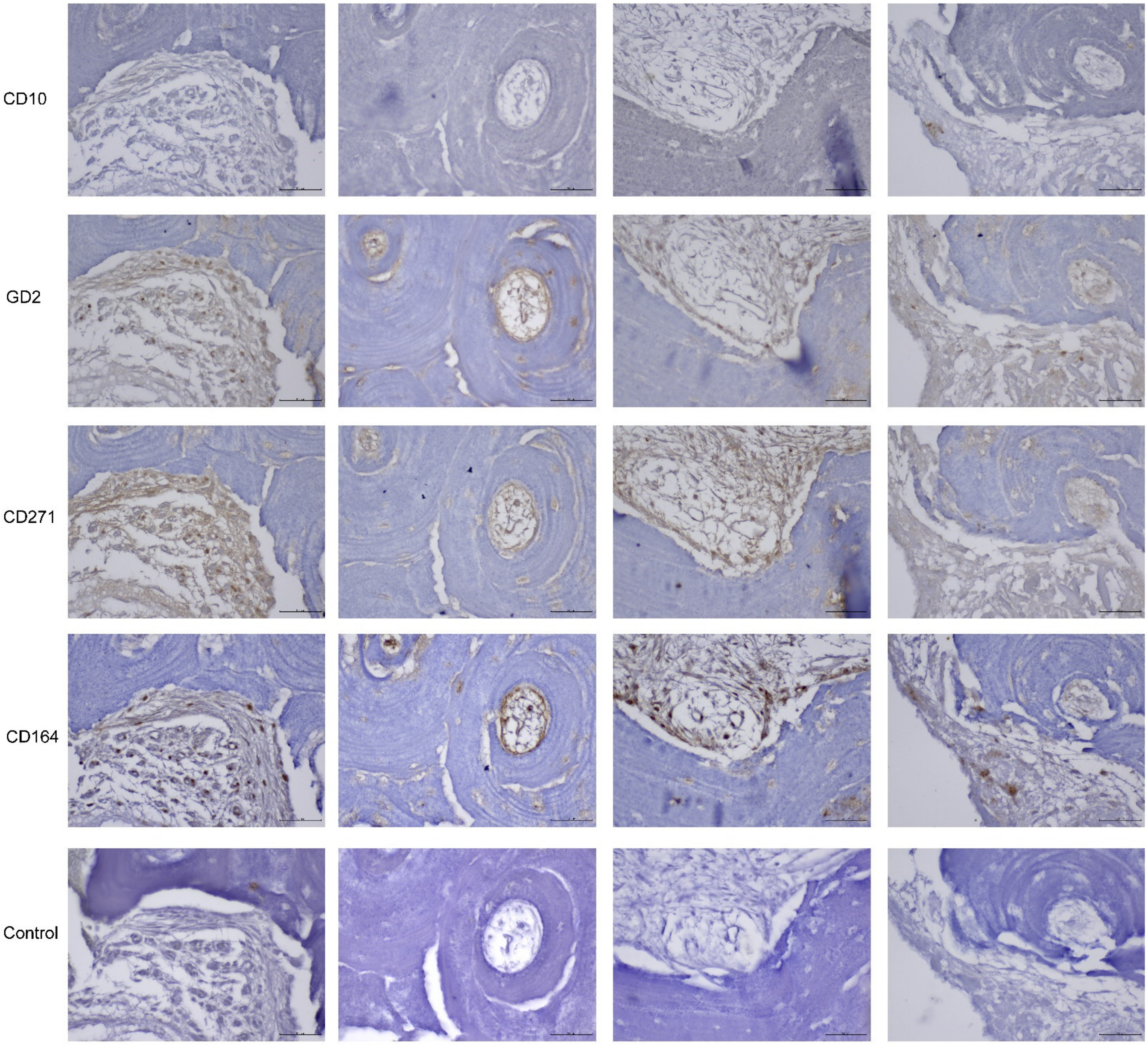

Figure S11. MSPC marker expression *in vivo* on primary hip osteoarthritis acetabulum samples from patient No 9.

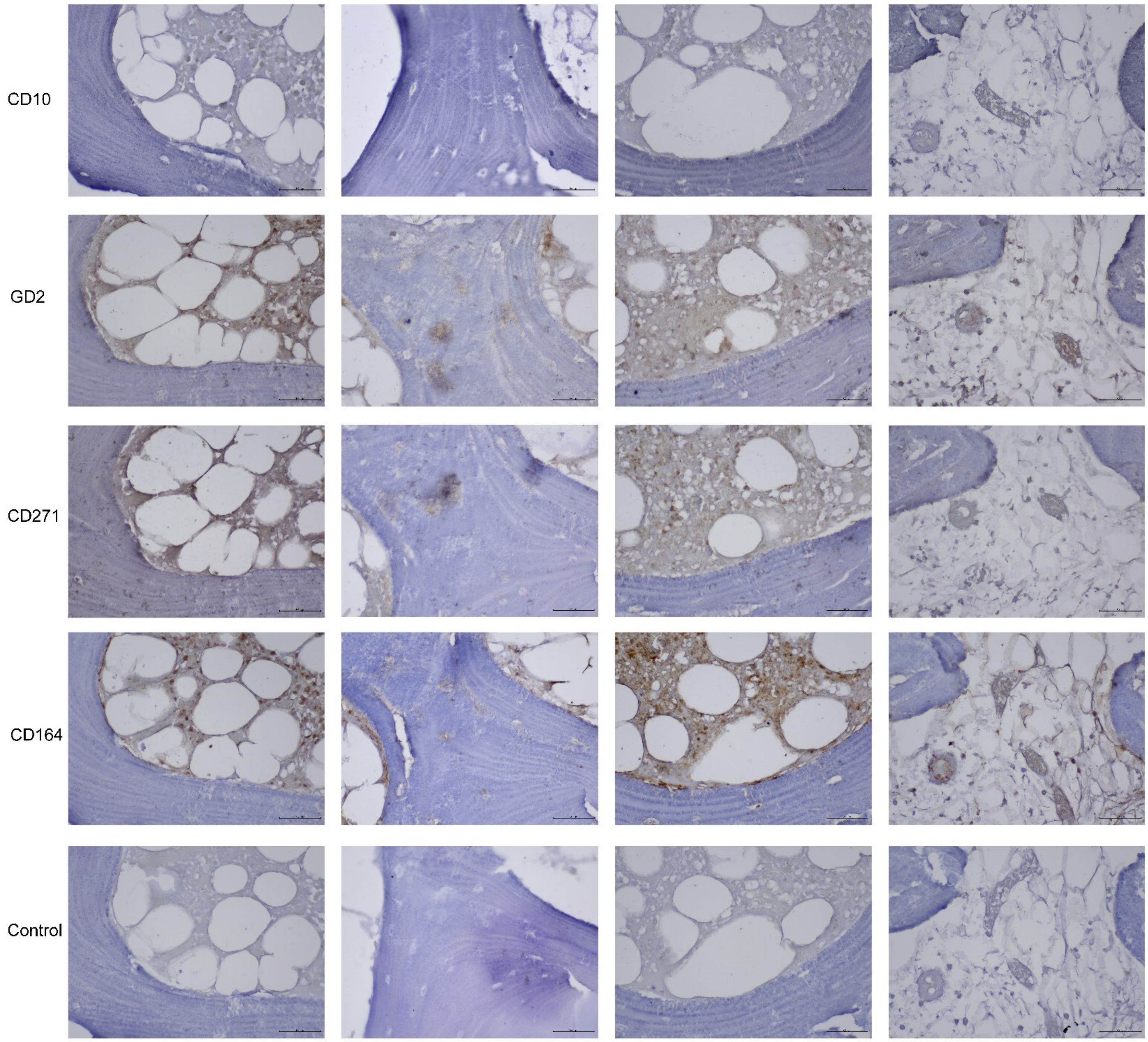

Figure S12. MSPC marker expression *in vivo* on primary hip osteoarthritis femoral head samples from patient No 9.

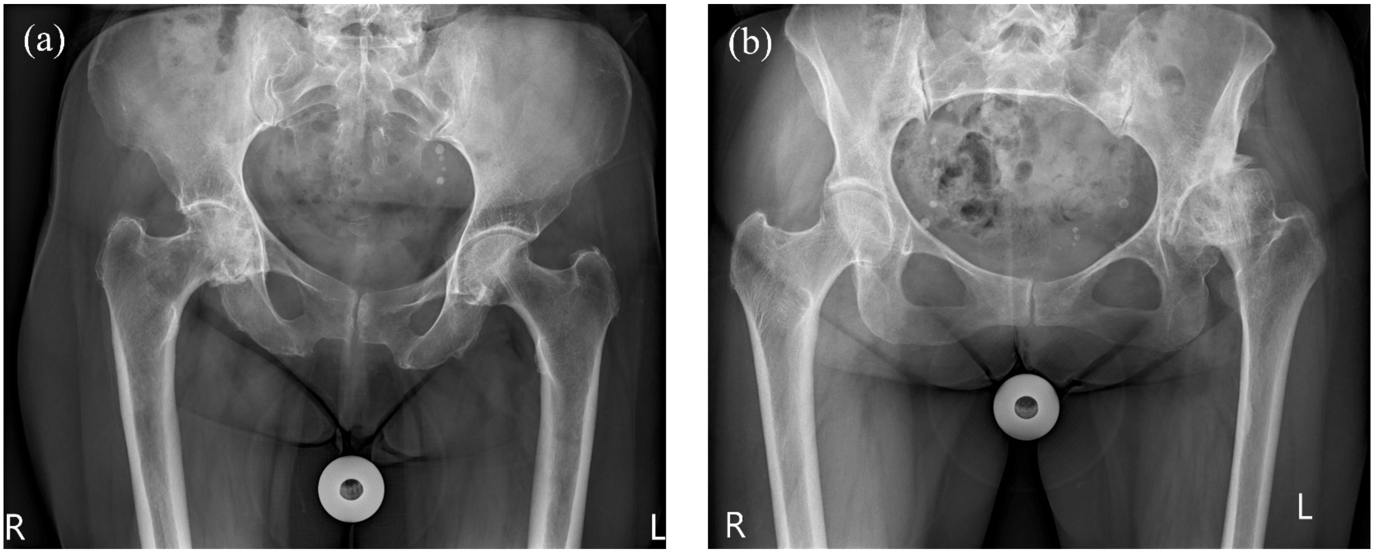

Figure S13. Preoperative anterior-posterior radiographs. **(a)** Anterior-posterior radiograph of a patient included in the primary hip osteoarthritis group. **(b)** Anterior-posterior radiograph of a patient included in the secondary osteoarthritis due to developmental dysplasia of the hips group.

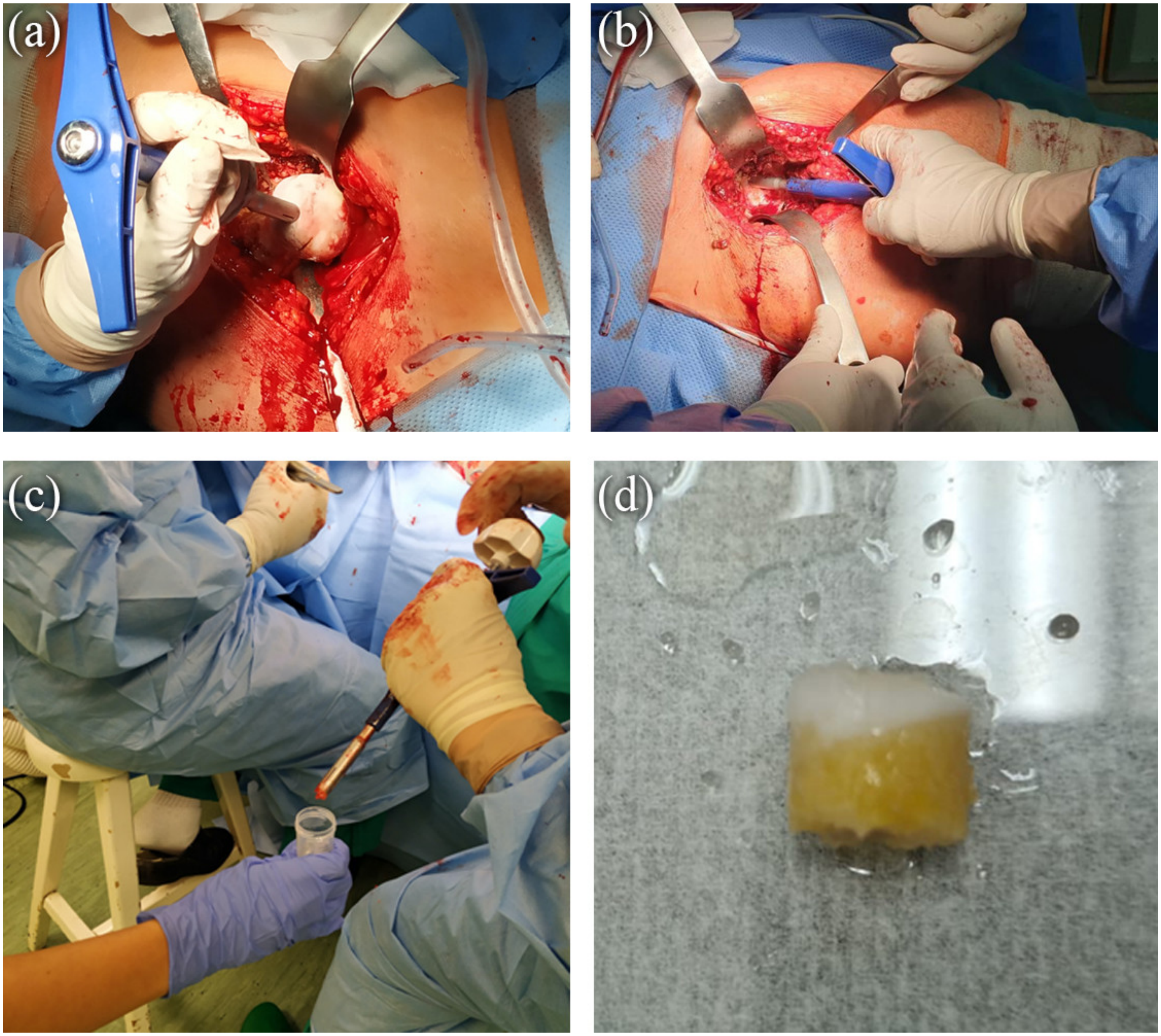

Figure S14. Presentation of how the samples were obtained. A) Obtaining a sample from the femoral head using a 10-mm diameter cylindrical chisel. B) Obtaining a sample from the acetabulum using a 10-mm diameter cylindrical chisel. C) Immediate transfer of the sample to a Falcon tube after harvesting. D) Example of one harvested sample in the laboratory before further procedures.

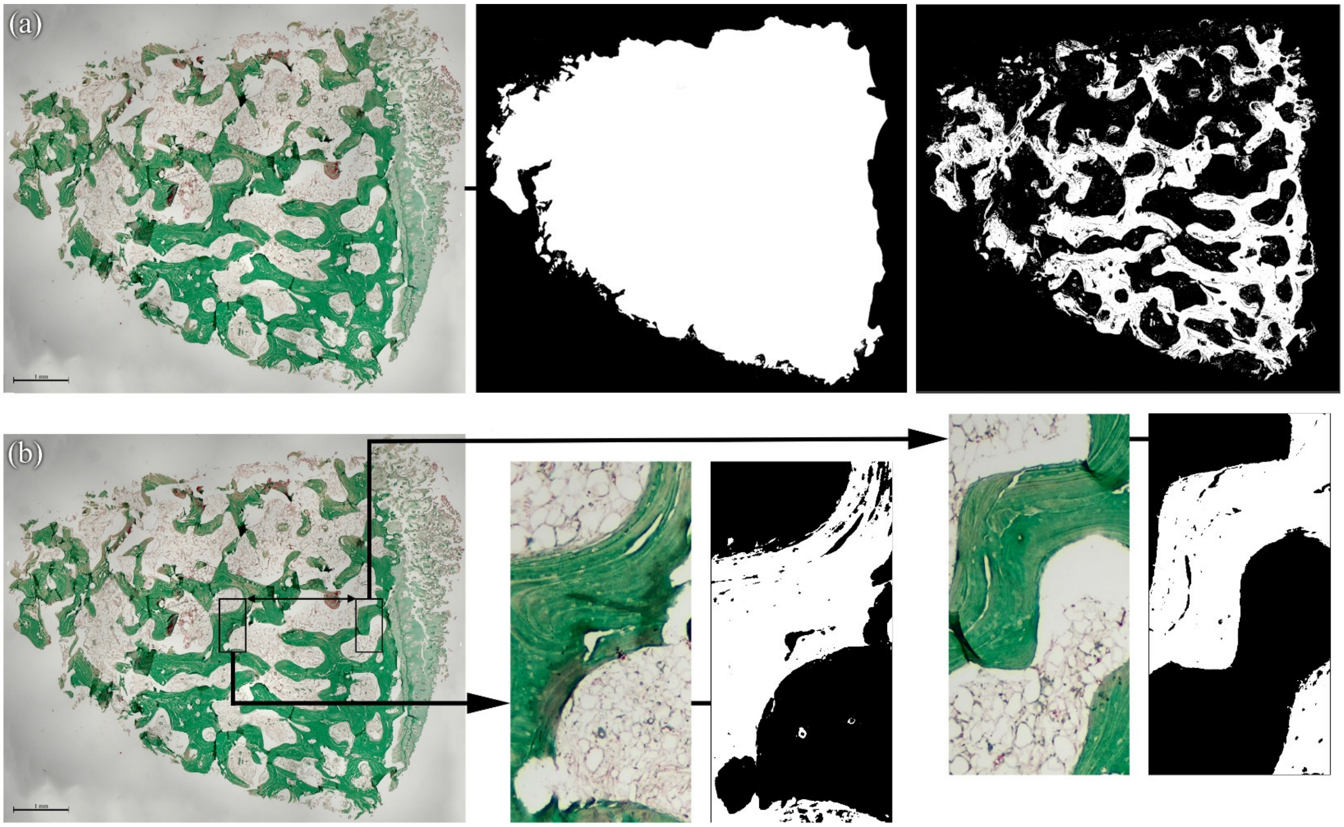

Figure S15. Histomorphometry. **(a)** First image shows Goldner staining of one Fh sample, the second image shows a mask that represents “Tissue Area (TA)”, while the third image shows a mask that represents “Bone Area (BA)”. These parameters are measured and divided, resulting in a total BA/TA (%).

(b) This image shows the same Goldner stained Fh sample in which a subchondral area is marked with a 500 x 1000 um box, and a box of the same size 2000 um distally. Both of these areas are measured using masks in a similar manner as for total BA/TA, resulting in Subchondral BA/TA (%) and Trabecular BA/TA (%).

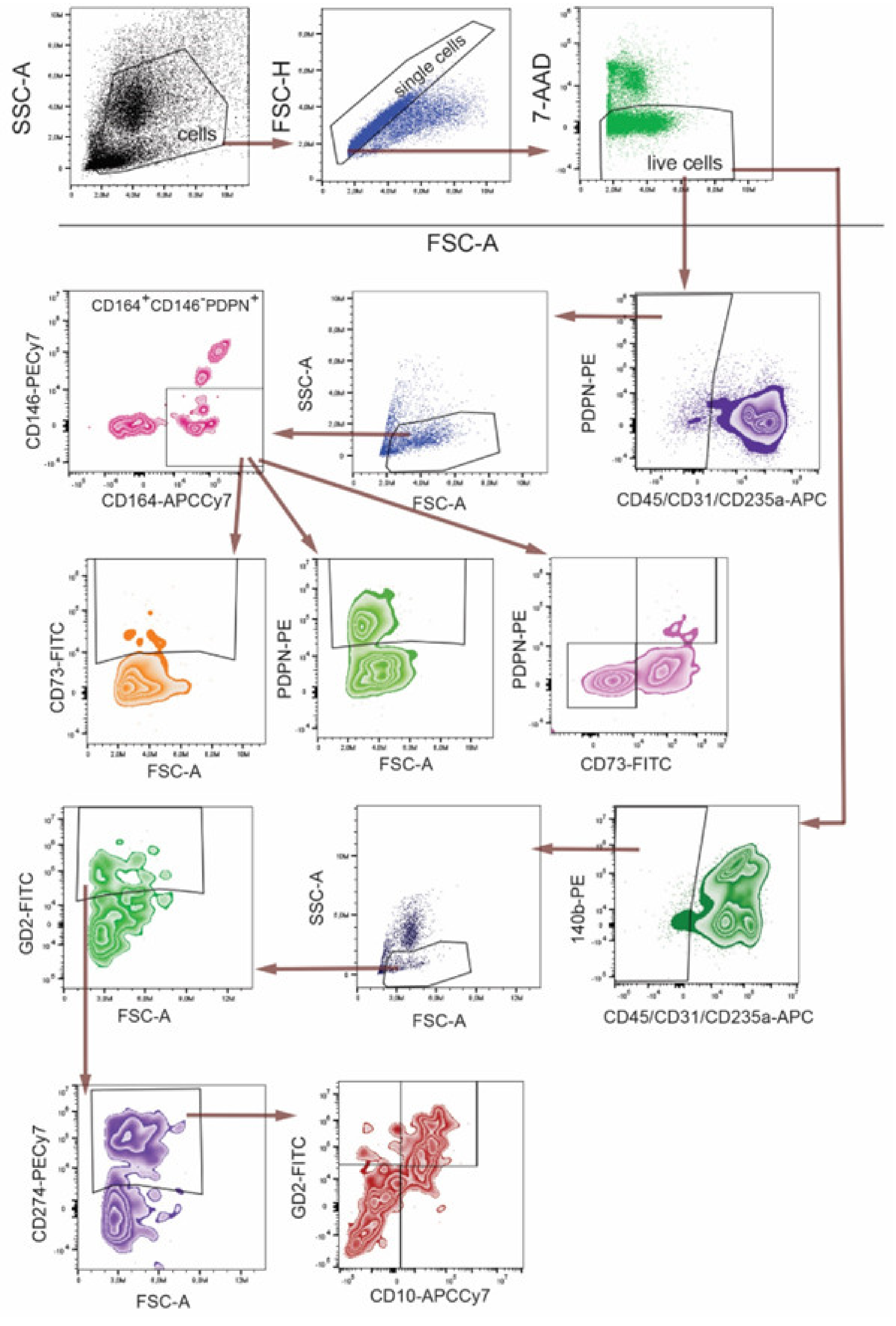

Figure S16. Schematics of flow cytometry analysis. Hematopoietic lineages were determined amongst single, live cells (7-AAD-). Non-hematopoietic cells were selected according to the absence of CD45, CD31and CD235a, and the remaining debris and aggregates were excluded again based on the scatter properties. Populations expressing progenitor markers were delineated according to the signals of non-stained cells and fluorescence minus one controls.
